# Novel Approaches to the Control of Oral Microbial Biofilms

**DOI:** 10.1155/2018/6498932

**Published:** 2018-12-31

**Authors:** Xinyi Kuang, Vivian Chen, Xin Xu

**Affiliations:** ^1^State Key Laboratory of Oral Diseases & National Clinical Research Center for Oral Diseases & Department of Cariology and Endodontics, West China Hospital of Stomatology, Sichuan University, Chengdu, Sichuan, 610041, China; ^2^Columbia University, New York, USA

## Abstract

Effective management of biofilm-related oral infectious diseases is a global challenge. Oral biofilm presents increased resistance to antimicrobial agents and elevated virulence compared with planktonic bacteria. Antimicrobial agents, such as chlorhexidine, have proven effective in the disruption/inhibition of oral biofilm. However, the challenge of precisely and continuously eliminating the specific pathogens without disturbing the microbial ecology still exists, which is a major factor in determining the virulence of a multispecies microbial consortium and the consequent development of oral infectious diseases. Therefore, several novel approaches are being developed to inhibit biofilm virulence without necessarily inducing microbial dysbiosis of the oral cavity. Nanoparticles, such as pH-responsive enzyme-mimic nanoparticles, have been developed to specifically target the acidic niches within the oral biofilm where tooth demineralization readily occurs, in effect controlling dental caries. Quaternary ammonium salts (QAS) such as dimethylaminododecyl methacrylate (DMADDM), when incorporated into dental adhesives or resin composite, have also shown excellent and durable antimicrobial activity and thus could effectively inhibit the occurrence of secondary caries. In addition, custom-designed small molecules, natural products and their derivatives, as well as basic amino acids such as arginine, have demonstrated ecological effects by modulating the virulence of the oral biofilm without universally killing the commensal bacteria, indicating a promising approach to the management of oral infectious diseases such as dental caries and periodontal diseases. This article aims to introduce these novel approaches that have shown potential in the control of oral biofilm. These methods may be utilized in the near future to effectively promote the clinical management of oral infectious diseases and thus benefit oral health.

## 1. Introduction

Oral biofilm, a structured community which consists of a wide range of microbes embedded with self-organized matrix of extracellular polysaccharides (EPS), is clearly recognized as a virulence factor to many oral infectious diseases including dental caries, gingivitis, periodontitis, periapical periodontitis and peri-implantitis [1–5]. Controlling oral biofilm incurs large expenditures worldwide [6]. With recent boom of new technologies and increased knowledge of genetic pathways, physiological responses, and intracellular signal transduction pathways, our understanding of biofilms has progressed significantly since they were first formally defined in the mid-1980s [7, 8]. Classical biofilm lifecycle includes bacterial attachment, biofilm growth/maturation, and biofilm dispersal. Measures that can disrupt any stage of biofilm cycle are considered as potential approach to the control of biofilm. Due to the complexity of the oral cavity and the rapid clearance of saliva, topically applied antibacterial agents are not retained at the proper concentrations for a long enough duration [9]. Compared with bacteria in planktonic, mature biofilm tends to need higher concentrations of antimicrobial agents to be eradicated [1, 10, 11]. When mixed with antimicrobial drugs, planktonic cells expose all cells to the full dose [12] while matrix in biofilm may reduce some drug access, preventing penetration of drugs into its deep layer [13–17]. Recent studies have shown that the EPS matrix not only provides functions as scaffold for biofilm growth and maturation but also provides emergent properties of biofilms including surface adhesion, spatial and chemical heterogeneities, synergistic/competitive interactions, and increased tolerance to antimicrobial agents [14, 18]. By contrast, little is understood about the most economic and effective ways of controlling oral biofilm due to the enhanced resistance to antibiotics and other antimicrobial agents [7, 16].

There have been increased attempts to develop ideal antimicrobial agents for the emergence of antibiotic-resistant bacteria [19]. The ideal antibiofilm approach is to facilitate the dispersion of formed biofilms, eliminate pathogens, and inhibit the formation of new biofilms while avoiding the elimination of commensals which may cause microecology dysbiosis [20–23]. Different from conventional antibiotics such as chlorhexidine, some of the novel treatment strategies for biofilm infections aim at specifically targeting unique biofilm characteristics [1] to minimize or eliminate the drug resistance of oral biofilm.

This review will introduce some of the novel strategies for the disruption/inhibition of oral biofilm, including nanomaterials, quaternary ammonium salts, small molecules, arginine, and the natural products.

## 2. Nanomaterials

Nanomaterials have revolutionized the concept of what a material is and can be since its discovery in the 1980s. Since then, nanomaterials have been employed in many fields including medicine and are projected to have broad prospects for future development [24]. Many nanomaterials, such as silver, copper oxide, zinc oxide nanoparticles, titanium oxide, and graphene, can be used to control biofilm formation [25–27]. Quaternary ammonium polyethylenimine, chitosan, and silica nanoparticles have also been suggested effective in controlling biofilms [25, 28, 29]. Moreover, applying nanomaterials for drug delivery, either as a carrier with specific affinity to tooth surfaces or as a drug for its inherent antimicrobial properties, have garnered attention in recent years [11, 28, 30, 31].

Certain metal nanomaterials show their capacities in controlling the oral biofilm. Among them, silver nitrate and silver nanoparticles (AgNPs) are the most effective against oral pathogens [32]. The drawback of silver nitrate is to cause dentine discoloration [5, 33, 34] whereas a silver nanocoating directly on dentine can successfully prevent the biofilm formation on dentine surfaces and inhibit bacterial growth in the surrounding media, suggesting a promising approach to protecting from dental plaque and secondary caries when applied as a dentine coating [5]. AgNPs exhibit the antibiofilm potential against* Enterococcus faecalis*, which is identified as the main cause of secondary and persistent endodontic infections [35, 36]. The application mode of AgNPs affects its antibiofilm efficacy. Using 0.02% AgNPs gel as medicament can significantly disrupt the structural integrity of the* E. faecalis* biofilm [37]. However, using AgNPs solution as an irrigant shows less effective against* E. faecalis *than NaOCl, which is commonly used in the endodontic treatment [38]. In addition, AgNPs could be a promising vehicle for calcium hydroxide as a short-term intracanal medicament to eliminate* E. faecalis* from human dentin [39].

Chitosan is a nontoxic natural cationic polysaccharide with characteristics of adhesiveness, antimicrobial activity, biocompatibility, and biodegradability [40, 41]. Because of its poor solubility above pH 6.5, chitosan exhibits its antibacterial activity better in an acidic condition [42]. Previous studies showed that chitosan inhibited the growth and adherence of* Streptococcus mutans* and other streptococci [43–45]. The chitosan nanoparticles (CNPs), although smaller than chitosan, still have the antimicrobial activity [42]. Owing to a higher surface charge density, CNPs could interact with the negative charge surface of bacterial cells, causing bacterial cell death [46]. CNPs, especially those prepared from low molecular weights chitosans, exhibit high antimicrobial effect towards* S. mutans* biofilm [47, 48]. CNPs inhibit other streptococci (such as* Streptococcus sobrinus*,* Streptococcus sanguinis* and* Streptococcus salivarius*) at low concentrations ranging from 0.312 mg/mL to 0.625 mg/mL [48]. Moreover, CNPs show inhibitory effect against* E. faecalis* and its biofilm [40, 49]. The commonly used intracanal medicament calcium hydroxide can damage the bacterial DNA due to its alkaline pH [50]. However,* E. faecalis* are alkali resistant and thus cannot be killed by calcium hydroxide in the infected root canal [51]. Adding CNPs into calcium hydroxide, the mixed intracanal medicament shows increasing antibacterial activity against* E. faecalis* and inhibits bacterial recolonization on root canal dentin compared with calcium hydroxide alone [52]. Microorganisms on the dental implants are the major causative factor of implant failure and peri-implantitis. The Ag-conjugated CNPs can inhibit the growth and adherence of* Porphyromonas gingivalis *and* S. mutans *and reduce the biofilm formation on dental implants, thus representing a prospective coating material for titanium dental implants [53].

Mesoporous silica nanoparticles (MSNs) have been used as biocatalysts, biosensors, drug delivery system, as well as imaging modality for diagnosis and therapy. In comparison with conventional nanoparticles, MSNs have the unique characteristics of mesoporous structure, large surface area and pore volume, stable physicochemical property, and flexible surface modification [54–56]. It has been reported that silica nanoparticles could inhibit adherence of bacteria [57]. Moreover, MSNs have the advantage of loading drug molecules with high capacity, well dispersity, costing less, relatively high biocompatibility, and available for custom design [55, 56]. MSNs, whether spherical or wiry, while loaded with antimicrobial agents such as chlorhexidine (CHX), can attach on microbes and release CHX up to 48 hours [58]. The MSNs-encapsulated-CHX (CHX@MSN) has demonstrated potent antibacterial activity against* S. mutans*,* S. sobrinus*,* Fusobacterium nucleatum*,* Aggregatibacter actinomycetemcomitans*, and* E. faecalis*, either in planktonic culture or in monospecies biofilms. It can also suppress multispecies biofilms of* S. mutans*,* F. nucleatum*,* A. actinomycetemcomitans* and* P. gingivalis* up to 72 h [59]. Addition of CHX@MSN to the dental filling materials such as glass ionomer cement (GIC) and resin composite can significantly inhibit the biofilm formation of* S. mutans* without compromise the mechanical properties of the filling materials [60, 61].

The “smart” drug delivery system is a delivery system of drugs by nanoparticles whose release is triggered by environmental stimuli such as pH, glucose or bacterial products [62]. Nanoparticles that are pH-responsive are stable at physiologic pH levels but degrade or disrupt at acidic pH levels to release the active drug [9]. Given that dental caries always occur at persistent low pH site around 4.5~5.5 on the teeth [63], where cariogenic organisms ferment sugar and create acidic niches, approaches to the control of cariogenic biofilms by targeting specific microenvironments have also been studied. This pH-responsive system makes full use of the acidic condition, with high affinity to hydroxyapatite, pellicle, and EPS surface, releasing drugs activated by low pH, where cariogenic bacteria prosper and actively develop biofilms. This delivery system can load up to ~22 wt% of farnesol, which is a hydrophobic antibacterial agent against planktonic* S. mutans*, but with limited activity against cariogenic biofilms. High affinity and capacity of this drug delivery system enhance the efficacy of farnesol, disrupt biofilms 4-fold more than free farnesol, and more importantly promote caries-reduction in rodent dental caries model [9].

Once inside the oral biofilm, bacteria are not easily disrupted, and it is therefore necessary to seek an approach that will disturb the matrix's integrity to eliminate the bacteria [64, 65]. Different from the nanoparticles with biological activity of antibacterial effects, catalytic nanoparticles (CAT-NP) can disrupt the matrix through its inherent enzyme mimic activity (e.g., peroxidase) when at acidic pH levels (greater catalytic efficiency at pH4.5-5.5, but minimal activity at neutral pH) [64]. Hence the catalytic nanoparticles are also termed nanozymes [66, 67]. Compared with other artificial enzymes that are based on organic molecules, CAT-NP possesses enhanced and versatile catalytic activities [68]. Hydrogen peroxide (H_2_O_2_) at proper concentration is commonly used as a disinfectant because it generates antimicrobial free radicals and degrades polysaccharides [65, 69]. CAT-NP can catalyze low concentrations (0.5–1%) of H_2_O_2_ in situ to simultaneously dissemble the biofilm EPS-matrix and kill embedded bacteria with high efficacy (>5-log reduction of cell viability; and >5000-fold more effective than H_2_O_2_ alone) [64]. Additionally, CAT-NP remains in the biofilm even after transient exposure [21]. CAT-NP is biocompatible because its catalytic activity is pH-dependent. At a physiological pH, free-radical production is minimized [64, 68]. No side effects to the oral mucosa tissue have been shown when CAT-NP is used* in vivo* with H_2_O_2_. In addition, CAT-NP can reduce apatite demineralization under acidic condition* in vitro* and thus attenuate the severity of carious lesions [64].

## 3. Quaternary Ammonium Salts (QAS)

Composite resin and adhesive system have been commonly used in clinical restoration for their esthetic effects [70–72]. However, nearly half of restorations fail within ten years [70, 72]. One primary reason is the development of secondary caries [72, 73], which is mainly caused by micro-leakage and dental plaque accumulation [74, 75]. So far, the mechanical properties and wear resistance of dental composites have been considerably improved while the antibacterial properties are still limited [75, 76]. Efforts have been made to inhibit secondary caries by adding antibacterial agents such as antibiotics and silver ions into the resin and adhesive systems [74, 77]. Polycations, such as quaternary ammonium salts (QAS), are of high molecular weight, non-volatile, chemically stable compared with conventional antibacterial agents [76], showing great potential to be added into the resin and adhesive system [78].

QAS, owing to their broad-spectrum of antimicrobial activity and low level of toxicity, were first used in mouthwash to control oral biofilm in the 1970s and later added into dental composite materials in the 1990s [79, 80]. The antibacterial mechanism of QAS is by binding their positive charge to the negatively charged bacterial cell membrane, causing lysis of cell membrane [76]. One type of QAS, quaternary ammonium dimethacrylate (QADM), has reactive groups on both ends of a dimethacrylate and can be incorporated in resin without compromising its mechanical properties [81].

12-methacryloyloxydodecyl-pyridinium bromide (MDPB), a QAS developed by Imazato et al., has shown strong antibacterial and antibiofilm effects against* S. mutans, E. faecalis*,* F. nucleatum*, and* Prevotella nigrescens* [82–84]. MDPB and methacryloxylethyl cetyl dimethyl ammonium chloride (DMAE-CB) can be incorporated into composites and inhibit the growth and adherence of oral pathogens [83–85]. Recent studies tend to mix QAS with other effective constituents to develop novel composites. A special QAS dimethacrylate monomer named ionic dimethacrylate monomers (IDMAs) can copolymerize with other methacrylate monomers (e.g., bisphenol A glycerolate dimethacrylate) and generate antibacterial polymers for dental composites and consequently reduce* S. mutans* colonization [86]. Composite with IDMA-1, when combined with calcium phosphate (CaP) particles and silver nanoparticles, demonstrates enhanced antibacterial activity with intact mechanical properties [87], indicating the potential of IDMAs in the development of novel antimicrobial composite resin.

Recently, new antibacterial monomers dimethylaminohexadecyl methacrylate (DMAHDM) and dimethylaminododecyl methacrylate (DMADDM) have been developed with enhanced antibacterial activity compared to QADM [88, 89]. The long chain polycations of these monomers bond to bacterial membrane is as a needle to a balloon [90]. DMADDM with a longer carbon chain length of 12 demonstrates stronger antimicrobial effect than DMAHM with a chain length of 6 [88]. It has been proven that the length of carbon chain can affect the antimicrobial efficacy [91, 92]. More importantly, DMADDM-containing adhesives showed an anticaries activity in a secondary caries animal model [93]. When DMADDM is incorporated with silver nanoparticles into adhesive system, it significantly reduces the metabolic activity of biofilm without affecting dentin bond strength [88]. Amorphous calcium phosphate (NACP) can neutralize acid attacks and release high levels of Ca and inorganic phosphate (Pi) ions, which promote tooth remineralization. Adding nanoparticles of DMADDM and NACP into composites and adhesive system results in a stronger antimicrobial potency, milder pulpal inflammation, and much more reparative dentin formation [94, 95]. Additionally, this NACP-and DMADDM-containing adhesive system possesses long-lasing antibacterial properties and strong bond strength [96]. Although the carbon chain length can affect antimicrobial efficacy, DMAHDM with a carbon chain length of 16 has a stronger antimicrobial effect than DMAODM with a chain length of 18 [91, 97, 98]. 10% DMAHDM of bonding agent can completely remove* S. mutans* biofilm* in vitro* [99]. Combination of DMAHDM with NACP into composites decreases biofilm metabolic activity, acid production, and the colony-forming units (CFU) of* S. mutans* [97].

Changing the functional group position of QAS may alter its anti-caries effects when incorporated into dental resin. Triethylaminododecyl acrylate (TEADDA), a new QAS with a different functional group position of DMADDM, when combined with adhesive resin, shows enhanced mechanical properties but reduced antibacterial effects compared with DMADDM [80].

However, frequent use of QAS may also lead to bacterial resistance [100]. CHX as the common disinfectant in mouthwash, has been proven to cause resistance in four oral bacteria species, i.e.,* Streptococcus gordonii, E. faecalis, F. nucleatum*, and* P. gingivalis *[101]. MDPB induced no resistance in* S. mutans *and* E. faecalis* [83]. Another study tested the bacterial resistance of eight oral bacteria (*S. mutans*,* S. sanguinis*,* S. gordonii*,* E. faecalis*,* A. actinomycetemcomitans*,* F. nucleatum*,* P. gingivalis*, and* Prevotella intermedia)* after treatment with DMADDM and DMAHDM and found that DMADDM induced resistance in only one species (*S. gordonii*), while DMAHDM induced resistance in none of the tested species [101].

## 4. Small Molecules

A novel strategy to control oral biofilm is to disrupt its formation [102]. Small molecules are promising for controlling biofilm formation due to their good stability, activity at low concentrations, and low toxicity [103].

**Figure 1:**
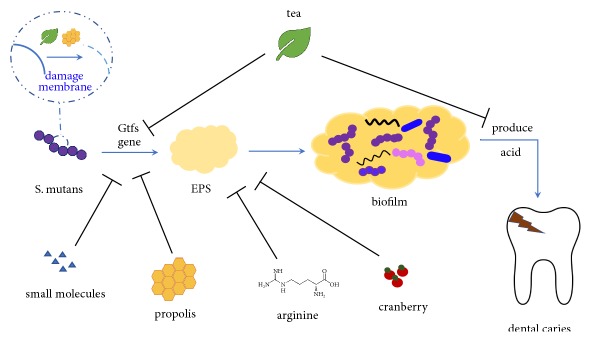
*S. mutans* is well recognized as the main cariogenic bacterium in the oral biofilm. It can metabolize carbohydrates to synthesize EPS as the scaffold of biofilm, produce acid accumulating at the biofilm/enamel interface to demineralize tooth hard tissue, and ultimately cause visible dental decay. Antimicrobial agents inhibit the aforementioned cariogenic process of* S. mutans* and may have the translational potential in the control of dental caries.

*S. mutans* is most widely regarded as the cariogenic bacterium in the oral biofilm. Though it may not be the most abundant, it can rapidly utilize dietary sucrose to synthesize EPS effectively, produce acid, and tolerate the acidic microenvironment in the cariogenic biofilm [104–106]. Three glucosyltransferases (GtfB, GtfC, and GtfD) of* S. mutans* have been identified, which synthesize adhesive EPS and contribute to the formation of cariogenic biofilms (Figure 1). Gtfs, specifically GtfB and GtfC secreted by* S. mutans,* can bind the pellicle formed on the tooth surface and produce glucans as specific binding sites for bacteria colonization [107, 108]. It has been proven that therapeutics aimed at interrupting the EPS synthesis by* S. mutans* are promising approaches to oral biofilm control [109], and glucosyltransferases may provide a good target for the inhibition of biofilm formation [110, 111]. GtfC is able to synthesizes both soluble and insoluble glucans [112, 113]. Lead compounds which target GtfC catalytic domain with high affinity and specificity have shown potential in controlling oral biofilm. Virtual screening from the small molecule library has identified two lead compounds, namely, #G43 and a quinoxaline derivative, 2-(4-methoxyphenyl)-N-(3-{[2-(4-methoxyphenyl) ethyl] imino}-1,4-dihydro-2- quinoxalinylidene) ethanamine. #G43 selectively inhibits the biofilm formation of* S. mutans* without disturbing the microecological balance between pathogens and commensal species (Figure 1) [110], while 2-(4-methoxyphenyl)-N-(3-{[2-(4-methoxyphenyl) ethyl] imino}-1,4-dihydro-2-quinoxalinylidene) ethanamine can inhibit the biofilm formation and promote the removal of mature biofilm of both* S. mutans* and* S. sanguinis*. More importantly, this small molecule shows good anticaries efficacy by significantly reducing the incidence and severity of smooth surface caries* in vivo* [111].

A group of synthesized small molecules are inspired by some natural products such as garlic, ursine triterpenes, ginseng, etc. These small molecules have good antibacterial and antibiofilm activity and induce low drug resistance [20]. One group of small molecules inspired by marine natural products, are derivatives based on the 2-aminoimidazole (2-AI) or 2-aminobenzimidazole (2-ABI) subunit [113, 114]. Eight of them are proved to inhibit* S. mutans in vitro* at a concentration lower than 4*μ*M without affecting the commensal bacteria. The most effective small molecule among them, 2A4, downregulates the expression of genes associated with biofilm and also inhibits the two key adhesins, antigen I/II and Gtfs, of* S. mutans* (Figure 1) [114]. 3F1, a compound in 2-aminoimidazole, can disperse mature* S. mutans* biofilm at 5*μ*M while not affecting the biofilm of* S. sanguinis* and* S. gordon*ii. Similarly to 2A4, 3F1 also targets key adhesins of* S. mutans*, and may represent a specific anticaries approach [115].

In addition, small molecules that target other oral pathogens have been developed.* Candida albicans *is a common commensal fugus in the oral cavity and can cause opportunistic fungal infection in susceptible populations [116]. Screening from a chemical library (NOVACore) has identified a unique series of diazaspiro-decane structural analogs that specifically targeted* C. albicans*. These compounds inhibit* C. albicans* filamentation and biofilm formation without affecting the growth of planktonic cells, making it a good candidate for the control of oral candidiasis [117].

## 5. Arginine

Frequent intake of dietary carbohydrates may lead to demineralization of tooth enamels by acidogenic bacteria in oral biofilm. However, periods of alkalization can promote remineralization to restore the integrity of the enamel [118]. Urea and arginine are two major substrates for alkali generation in oral biofilm. Some commensals are able to produce alkaline compounds to counter the acid stress imposed by acidogenic bacteria such as* S. mutans* and maintain a healthy oral biofilm [16, 119]. Oral commensals produce alkali mainly through the arginine and urea metabolism pathways [16]. A prevailing route of arginine metabolism is the arginine deiminase system (ADS), yielding ornithine, ammonia, CO_2_ and ATP [16, 118]. Oral* streptococci*, including* S. sanguinis*,* S. gordonii*,* S. parasanguinis*,* S. intermedius*,* S. cristatus*, and* S. australis*, certain* Lactobacillus* species, and a few* spirochetes* can express ADS [118]. Using arginine-containing toothpaste can significantly increase ADS activity in plaque of caries-active individuals, shifting the bacteria composition to one similar to that of caries-free individuals [120, 121]. Therefore, increasing the availability of exogenous arginine in the oral environment could be a novel approach to controlling biofilms.

Arginine as a natural dietary supplement has shown good activity against bacteria growth, virulence, coaggregation, and biofilm formation [122]. An arginine-rich polycationic protein, protamine, can inhibit oral pathogens such as* A. naeslundii*,* A. odontolyticus*,* E. faecalis*,* Lactobacillus acidophilus*,* F. nucleatum*,* P. gingivalis*,* C. albicans*, and* A. actinomycetemcomitans* [123]. Arginine suppresses the production and composition of extracellular membrane glucans, thereby inhibiting the adherence activity of* S. mutans* to the tooth surface [124]. L-arginine is able to reduce the biomass of polymicrobial dental biofilms, particularly inhibit the biofilm formation of* S. mutans* by reducing its water-insoluble EPS production (Figure 1) [119, 125]. A recent study showed that 1.5% L-arginine enriched* S. gordonii *while suppressing* S. mutans*. The arginine-treated biofilm exhibited significantly higher pH values at the biofilm-sHA interface [119]. In addition, L-arginine was shown to inhibit the coaggregation of* P. gingivalis* and* Prevotella oris in vitro* [126]. Addition of 7% arginine to the dental adhesives enhanced biofilm formation without compromising its mechanical properties [127]. More importantly, combinatory use of arginine with NaF could synergistically inhibit* S. mutans* but enrich* S. sanguinis* while suppressing the overgrowth of* P. gingivalis* in a multispecies biofilm, offering an ecological approach to the control oral biofilm [128].

## 6. Natural Products

Natural products, though its structure may be uncertain, exhibit biological activities that make them promising to be alternative or adjunctive therapies towards oral biofilm [129]. Polyphenols, which are defined as any substance that contains at least one aromatic ring with one or more hydroxyl groups and other substituents, have been identified as active compounds in many natural products such as tea, propolis, cranberry,* Galla chinensis*, grapes, coffee, and cacao polyphenols [130–132].

### 6.1. Tea

Tea (*Camellia sinensis*) has many health benefits including antioxidant, antimutagenic, antidiabetic, hypocholesterolemic, antibacterial, anti-inflammatory, and cancer-preventive properties [133–135]. Distinguished by the processing methods, there are three major types of tea: green tea (nonfermented), oolong tea (semifermented) and black tea (fermented) [136]. The anticaries effect of tea has been well suggested for decades. Using mouth wash containing green tea extract three times a day for a week could reduce salivary level of* S. mutans* and* Lactobacilli* [137]. The extracts of tea could inhibit the growth [138, 139], adherence [140], and acid production of the acidogenic oral* streptococci* [140, 141].

Although tea is rich in fluoride which is beneficial to caries prevention, its activity against oral biofilm formation is mainly attributed to polyphenols [133, 138, 139, 142, 143]. Tea catechins are polyphenols in green tea which have been recognized as the main antimicrobial components against oral pathogens [144, 145]. Tea polyphenols consist of (+)-catechin (C), (-)-epicatechin (EC), (+)-gallocatechin (GC), (-)-epigallocatechin (EGC), (-)-epicatechin gallate (ECg), (-)-epigallocatechin gallate (EGCg), (-)-catechin gallate (Cg), and (-)-gallocatechin gallate (GCg) [144]. EGCg and ECg and ECg are the most abundant and active tea catechins [140, 144, 146], possibly due to the presence of galloyl groups [147]. The antimicrobial mechanism of tea catechins is the irreversible damage to the microbial cytoplasmic membrane (Figure 1) [144]. In addition, tea catechins also inhibit the activity of salivary amylase, leading to reduced cariogenicity of starch-containing foods [148, 149]. Xu et al. demonstrated that EGCg in green tea at sub-MIC levels suppressed multiple cariogenic virulence factors of* S. mutans* associated with carbohydrate metabolism and acid tolerance, thus promoting the control of dental caries (Figure 1) [150]. Lee et al. verified that EGCg in green tea was a powerful antimicrobial agent against planktonic* E. faecalis *and its biofilm, being able to suppress expression of genes related to its virulence and biofilm formation [151]. In addition, EGCg could also inhibit GtfB/C/D genes of* S. mutans*, and thus suppressed the biofilm formation of this cariogenic bacterium (Figure 1) [152].

Periodontitis is a multifactorial chronic infectious disease that affects periodontal tissue. Bacteria are the primary etiological factor of periodontal diseases. Periodontitis is closely associated with a special group of Gram-negative anaerobic bacteria (mainly* P. gingivalis*,* Treponema denticola*, and* Tannerella forsythia*) that interact with tooth supporting tissues and immune cells [153, 154]. An epidemiological study showed that frequent consumption of green tea was positively correlated with good periodontal health [155]. Consistently,* in vitro* studies showed EGCg and ECg in green tea could significantly inhibit the growth, adherence, and biofilm formation of* P. gingivalis* [156, 157], suppress the activity of collagenase [147] and MMPs [158], and enhance gingival keratinocyte integrity to protect from invasion of* P. gingivalis* [159]. Besides, EGCg can inhibit another periodontal pathogen,* F. nucleatum*, by decreasing the adherence and the biofilm formation of this bacterium [160]. Interestingly, EGCg can potentiate the effect of conventional antibiotics (metronidazole, tetracycline) which are used in periodontal therapy [161]. Moreover, tea catechins are able to reduce halitosis which is associated with volatile sulfur compounds (VSCs) produced mainly by oral anaerobes such as* P. gingivalis* and* F. nucleatum* [162]. Using mouth wash containing tea catechins for 4 weeks could reduce halitosis [163]. Yasuda et al. showed that EGCg could remove CH_3_SH, a major component of VSCs, through a chemical reaction in the presence of oxygen [164]. In addition, Xu et al. proved that EGCg inhibited CH_3_SH production by suppressing* mgl* gene expression of* P. gingivalis* [165].

In addition to its antibacterial activity, EGCg exhibits inhibitory effect on the growth and hyphal formation of* C. albicans* [166, 167],  , and it can synergize with antimycotics against* C. albicans* biofilm [166, 168].

### 6.2. Propolis

Propolis is a hard, resinous, nontoxic natural product sourced from plants with a history of being used as a dietary supplement [169]. Ethanolic extracts of propolis (EEPs) have shown inhibitory effect against the growth and the adherence of* S. mutans* [170–173] and a comparable inhibitory effect to CHX when towards* Lactobacilli*,* P. intermedia*,* P. gingivalis*,* A. israelii*, and* C. albicans* [174]. Additionally, the anticaries effect of propolis was proven in desalivate rats [173, 175]. Among several compounds in propolis, flavonoid and cinnamic acid derivatives are considered to be the main bioactive constituent against bacteria [169]. Intriguingly, extracts from a novel type of propolis, which contain no traces of flavonoids and cinnamic acid derivatives, also show inhibition on Gtfs and growth and adherence of* S. mutans* due to its bioactive fraction containing a high abundance of fatty acids [176, 177].

Koo et al. identified two compounds, apigenin and trans-trans farnesol (tt-farnesol), which exhibited distinct biological activities against dental caries without impacting on bacterial viability [178, 179]. Apigenin, a 4*β*, 5, 7-trihydroxyflavone, is proven to effectively inhibit Gtfs, specifically GtfB and C (Figure 1). tt-farnesol is promising to be a novel adjunctive natural anticaries agent, as it is the most effective antibacterial compound in propolis. It is reported to reduce cell viability by disrupting membrane integrity and destabilizing the oral biofilm rather than affecting Gtfs activities (Figure 1) [178, 180]. Moreover, tt-farnesol can reduce the severity of smooth surface caries in rats [179]. The mechanism of tt-farnesol is attributed to the lipophilic moiety interaction with bacterial membrane, which is consistent with reduction of IPS accumulation in tt-farnesol treated* S. mutans* biofilms [179, 181]. One study examined the effect of topical applications (twice a day, 1 min exposure) of 1 mM apigenin, 5 mM tt-farnesol, and 13 mM fluoride (equivalent to 250 ppm F), alone or in combination, on the formation of* S. mutans* biofilms. The combination of the three shows the most effective impact on reducing the biofilm and acidogenicity of* S. mutans* [182]. Recently, Franca et al. designed a novel varnish with a propolis and chitosan base. This varnish adheres to the tooth surface, quickly forms film on the tooth surface, and continuously releases propolis for more than one week. The antimicrobial activity of the varnish against oral pathogens is similar to or even better than chlorhexidine varnish [183].

### 6.3. Cranberry

Cranberry is a highly nutritious fruit which is rich in a variety of bioactive compounds including flavonols, anthocyanins, tannins, flavan-3-ols and the phenolic acid derivatives [184]. Known for its high concentration in total polyphenols, the cranberry has been recognized as an excellent antioxidant and is beneficial to fighting bacterial infection [185]. It has well documented that cranberry is effective against oral infectious diseases, urinary tract disorders, cardiovascular diseases, and cancer [186, 187]. It has also shown antimicrobial activity against pathogens such as* Helicobacter pylori*,* Salmonella*,* Staphylococcus aureus*, and* Escherichia coli* [184]. Cranberries have demonstrated potential inhibitory effects against bacteria related to dental caries and periodontal diseases [188]. A cranberry-containing mouthwash could reduce the* S. mutans* counts after daily use for 6 weeks in a preliminary human trial [189]. Proanthocyanins (PACs) and flavonols are the most active components of the cranberry that can disrupt biofilm formation of* S. mutans* [190–192]. The highly purified A-type PAC (at 1.5 mg/ml) reduces biofilm formation of* S. mutans* and diminishes the acidogenicity of* S. mutans* even with lack of bactericidal effect [193]. Kim et al. also verified that topical application of PACs oligomers (100–300 *μ*M) with myricetin (2 mM) of cranberry twice a day to cariogenic biofilm could break down its microarchitecture, reduce the amount of insoluble EPS (Figure 1), and increase the pH values at the biofilm-apatite interface [194]. Inhibition of biofilm formation is ascribed to PACs prevention of bacterial coaggregation, reduction of bacterial hydrophobicity, and alternation of cell surface molecules [195, 196]. Investigation of the degree-of-polymerization (DP) of PACs oligomer reveals that DP 4 and DP 8 to 13 are the most effective in disrupting bacterial adhesion to glucan-coated apatite surfaces [197]. PACs are more effective in reducing the development of carious lesions on the smooth surface than on the sulcal surface, but less effective than fluoride (at 225–250 ppm)* in vivo* [193]. Evidently, PACs of cranberry are promising novel alternatives or adjunctive anticaries chemotherapy [198].

PACs are also effective for the prevention and management of periodontitis. The A-type PAC reduces the biofilm formation, adherence and invasiveness to the human epithelial cells and proteinase activity* P. gingivalis* [199, 200]. Bodet et al. verified that the constituents of cranberry could inhibit metalloproteinases and elastase induced by lipopolysaccharide (LPS)* in vitro *[201]. La et al. proved that the A-type PAC of cranberry could inhibit MMP-1, -3, -7, -8, -9, and -13 production by LPS-stimulated macrophages [202] and decrease the secretion of chemoattractant such as IL-8 and CCL5 [199].

## 7. Conclusion and Future Prospects

Because of drug resistance, more attention is being drawn to identify alternative agents to biofilm control. The precision, effectiveness and efficiency of targeting oral biofilm are emphasized. Novel nanomaterials, which have the ability to load antimicrobial drugs or act as the drugs themselves, can precisely target the pathogen in response to specific environmental stimuli. QAS, which can be incorporated into the dental composite resin and adhesive system, exhibits excellent antibacterial activity to prevent secondary caries. Custom-designed small molecules that target key factors mediating bacterial adherence are also promising agents to disrupt oral biofilm. Arginine, as a base-generation substrate of oral bacteria, can function as an eco-modulator of oral biofilm and thus prevent dental caries. Furthermore, a group of natural products which contain polyphenols possess antimicrobial and antibiofilm activity with low drug tolerance towards oral biofilm.

However, it is noteworthy that current data available are mostly obtained from* in vitro *or animal studies using single species biofilm. The polymicrobial infection nature of dental caries and periodontitis would limit the clinical translation of the approaches developed based on single species biofilm. The complex environment in the oral cavity, particularly rapid clearance by saliva, will also affect the bioavailability and subsequently the effectiveness of the novel agents* in vivo*. More studies are needed to further evaluate the antimicrobial activities in humans to balance the bioactivity and biocompatibility of the novel agents as well.
